# Multiline Laser Interferometry for Non-Contact Dynamic Morphing of Hierarchical Surfaces

**DOI:** 10.3390/biomimetics10080486

**Published:** 2025-07-23

**Authors:** Biagio Audia, Caterina Maria Tone, Pasquale Pagliusi, Alfredo Mazzulla, George Papavieros, Vassilios Constantoudis, Gabriella Cipparrone

**Affiliations:** 1 Department of Physics, University of Calabria, Ponte P. Bucci 31C, 87036 Rende, CS, Italy; biagio.audia@unical.it (B.A.); pasquale.pagliusi@fis.unical.it (P.P.); 2 CNR Nanotec, Institute of Nanotechnology, S.S. Cosenza, 87036 Rende, CS, Italy; caterinamaria.tone@cnr.it (C.M.T.); alfredo.mazzulla@cnr.it (A.M.); 3 Institute of Nanoscience and Nanotechnology, NCSR Demokritos, 15341 Agia Paraskevi, Greece; g.papavieros@inn.demokritos.gr (G.P.); v.constantoudis@inn.demokritos.gr (V.C.); 4 Nanometrisis P.C, 15341 Agia Paraskevi, Greece

**Keywords:** polychromatic vectorial interferometry, surface photopatterning, optical Fourier surface, hierarchical topography

## Abstract

Hierarchical surface structuring is a critical aspect of advanced materials design, impacting fields ranging from optics to biomimetics. Among several laser-based methods for complex structuring of photo-responsive surfaces, the broadband vectorial interferometry proposed here offers unique performances. Such a method leverages a polychromatic laser source, an unconventional choice for holographic encoding, to achieve deterministic multiscale surface structuring through interference light patterning. Azopolymer films are used as photosensitive substrates. By exploring the interaction between optomechanical stress modulations at different spatial periodicities induced within the polymer bulk, we demonstrate the emergence of hierarchical Fourier surfaces composed of multiple deterministic levels. These structures range from sub-micrometer to tens of micrometers scale, exhibiting a high degree of control over their morphology. The experimental findings reveal that the optical encoding scheme significantly influences the resulting topographies. The polarization light patterns lead to more regular and symmetric hierarchical structures compared to those obtained with intensity patterns, underscoring the role of vectorial light properties in controlling surface morphologies. The proposed method is fully scalable, compatible with more complex recording schemes (including multi-beam interference), and it is applicable to a wide range of advanced technological fields. These include optics and photonics (diffractive elements, polarimetric devices), biomimetic surfaces, topographical design, information encoding, and anti-counterfeiting, offering a rapid, reliable, and versatile strategy for high-precision surface structuring at a submicrometric scale.

## 1. Introduction

Nature has long served as an inspiration for technological advancements, particularly in the design and fabrication of functional surfaces. The hierarchical micro- and nano-patterned structures found in biological organisms exhibit remarkable properties such as adhesion control, self-cleaning, antifouling, optical effects, and enhanced mechanical performances [1,2,3,4,5,6,7,8,9,10,11]. Mimicking these natural designs has led to the development of biomimetic surfaces with broad applications in engineering, medicine, energy, and beyond [1,2].

To meet the increasing demand for highly precise micro- and nano-structured surfaces with complex and hierarchical configurations, various fabrication techniques have been developed [11,12,13,14,15,16,17,18,19]. Conventional lithographic techniques, including electron beam, nanoimprinting, and scanning probe lithographies, enable precise patterning at the micro- and nanoscale [15,16,17,18,19,20]. However, these methods often require multi-step or post-processing treatments, are time-consuming, and lack scalability for large-area structuring. Alternative methods such as chemical etching, electrostatic spraying, and brazing offer viable approaches but frequently struggle with reproducibility and environmental sustainability [12,14].

Among the various structuring techniques, optical processing methods stand out due to their high spatial resolution, non-contact nature, and ability to achieve high-fidelity patterning. In particular, laser-based fabrication techniques [15], such as Direct Laser Writing and Direct Laser Interference Patterning, have demonstrated remarkable capabilities in generating bio-inspired hierarchical structures [11,13,16,17,18,19]. These approaches leverage the interplay between laser parameters (such as intensity, polarization, and incidence angle) and material response, enabling the formation of complex periodic topographies with multiple functionalities [11,19].

Laser interference patterning, in particular, exploits the interference of coherent laser beams to produce well-defined light structures enabling periodic surface patterning [21,22,23,24]. By carefully controlling the intensity and polarization of the interfering beams, it is possible to tailor the geometry and anisotropy of the generated topographies [25,26,27]. This method enables large-area structuring with sub-micrometer resolution and has been successfully applied in fields such as photonics, biomaterials, and wettability engineering [24].

Morphing soft photoresponsive materials via laser interferometry provides a compelling strategy for achieving sinusoidal surface modulations and even complex Fourier surfaces [13,25,26,28]. Moreover, it has been demonstrated to be a method capable of dynamically modifying surface geometries and suitable for developing reconfigurable optics.

Azomaterials, for example, have been largely explored for large-area all-optical structuring, as the surface relief formation occurs through directional mass migration along the grating wave vector, driven by the light intensity or polarization patterns [22,29,30,31]. When reversible processes are involved as in the case of azobenzene-based materials, active and dynamic topographical modification can be easily achieved [30,32].

The reconfiguration of complex microtextures by light patterns with high precision offers tunable functionalities, such as controlled wettability and tailored diffraction properties, through simple and dynamic adjustments of the illumination parameters. Importantly, the reversibility of this process, as well the non-contact, remote control of the light patterning, preserves opportunities for further modifications, making it a highly versatile and scalable approach for applications requiring dynamic and customizable surface functionalities.

To further expand the capabilities of holographic encoding for complex and multifunctional surface textures, various multiplexing techniques, including sequential or multiple exposures to different light sources and stimulus-combined solutions, have been investigated [33,34].

Here, we report a multiline holographic approach we recently developed, which enables the single-step inscription of hierarchical Fourier topographies onto the free surface of an azobenzene-based polymer. This method leverages the interference of two polychromatic beams, comprising closely spaced wavelengths from a common laser cavity. By carefully regulating operational conditions and broadband tunable optics, it is possible to adjust independently the number of laser lines, their relative intensities, and their polarization states, thus allowing for the design of customized spatio-spectral light patterns. This capability holds great promise for advancing the field of biomimetic and reconfigurable optical surfaces, with potential applications spanning bio-environments, optoelectronics, wettability control, and photonics.

## 2. Multiline Laser Interferometry

**Figure 1:**
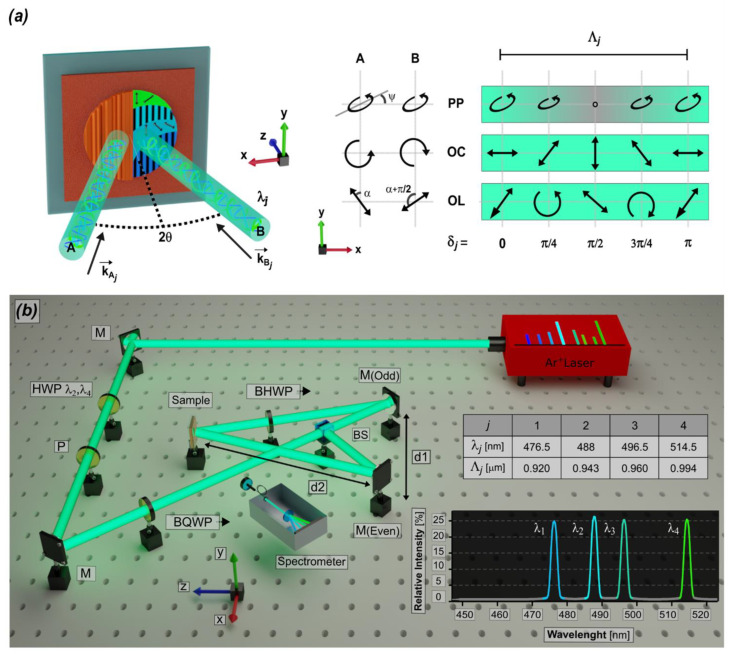
**Multiline surface photo-patterning.** (**a**) Schematic representation of the recording geometries in the *xyz* laboratory frame. In the right part, schematics of the light patterns for intensity (PP) and polarization (OC and OL), characterized by a spatial periodicity, Λj; the phase term δ_j_ = (2πxsinθ)⁄λ_j_ = πx⁄Λ_j_ represents the half-phase difference between the two recording beams at wavelength λ_j_. (**b**) Sketch of the experimental setup. An Ar^+^ laser beam is firstly directed through a filtering system, composed of a half waveplate (HWP) and a polarizer (P); broadband waveplates (BQWP and BHWP) are exploited along the path to independently set the intensities and the polarization states of the different lines. A non-polarizing beam splitter (BS) cube enables us to set the two beams with equal intensity which are then overlapped on the sample via a couple of metallic mirrors. The distance d1 between the two mirrors is 12 cm, while the distance d2 between the mirrors M(Odd), M(Even) and the sample is 23 cm. The two multiline beams cross at an angle θ with the *z*-axis, generating the light patterns in the *xy* plane. In the table, the four wavelengths (λ*_j_*) of the laser beam, and the corresponding spatial periodicities (Λ*_j_*) evaluated for an angle *θ* = 14.5°.

The proposed interferometric methodology for designing complex topography with hierarchical structures is based on the use of a laser operating in multiline configuration to generate spatio-spectral light patterns by the superposition of two light beams with a Gaussian profile (Figure 1). The stability condition of the light structures is relevant during the photo-morphing process since it ensures a high-fidelity recording of the designed light patterns at the micro- and nanoscale. Therefore, the single laser cavity of the multiline beam approach ensures a high correlation of the optical fields, optimum spatial overlap conditions, and extremely high pattern stability. Indeed, the light waves at each wavelength share the same geometric parameters and coherence length. Moreover, differently from multi-source holographic procedures, each line here traverses identical ray paths and experiences the same fluctuations.

The laser source we used for experimental studies is an argon ion laser (Innova 90C, Coherent Inc.) that generates a light beam with up to eight wavelengths, ranging from 457.9 nm to 514.5 nm, and a total power whose maximum value reaches 4.5 W. Adjusting the current in the Ar^+^ tube allows selective lasing over a defined number of spectral lines (denoted as *j*), effectively shaping the beam’s spectral profile. The relative intensities of these lines, governed by parameters such as gas pressure, discharge current, and inter-line gain competition, are further modulated using a filtering system (F). This system incorporates half-wave plates (HWPλ_3_, λ_6_) positioned before a vertically oriented polarizer (P), as shown in Figure 1. By incorporating broadband quarter-wave and/or half-wave plates along the optical paths of the interfering beams, it becomes possible to uniformly adjust the polarization state of each spectral line, enabling consistent vectorial patterns (e.g., intensity or polarization distributions, Figure 1a). For even finer spectral control, the polarization state of each line can be individually tuned using a broadband, electrically adjustable waveplate based on liquid crystal technology, the operating principles of which are detailed in [35].

Multiple and mixed light patterns can be designed as simple two beams interference. If we consider the two interfering beams as plane waves, in the paraxial approximation, the doable interference patterns can be described by the following Stokes vector (S_R_):

(1) $$S_{R}=\sum_{j}^{PP}\frac{I_{j}}{2}\left(1+cos2\delta_{j}\right)\left(\begin{array}{c} 1\\ \begin{array}{c} cos2\chi_{j}\;cos2\psi_{j}\\ \begin{array}{c} cos2\chi_{j}\;sin2\psi_{j}\\ sin{2\chi }_{j} \end{array} \end{array} \end{array}\right)\;+\sum_{j}^{OC}I_{j\;}\left(\begin{array}{c} 1\\ \begin{array}{c} cos2\delta_{j}\\ \begin{array}{c} -sin2\delta_{j}\\ 0 \end{array} \end{array} \end{array}\right)+\sum_{j}^{OL}I_{j\;}\left(\begin{array}{c} 1\\ \begin{array}{c} cos2\delta_{j}\;sin2\alpha \\ \begin{array}{c} cos2\delta_{j}\;cos2\alpha \\ sin2\delta_{j}\;cos2\alpha \end{array} \end{array} \end{array}\right)$$

where δ_j_ = (2πx sinθ)⁄λ_j_ = πx⁄Λ_j_ and Λ_j_ represents the half phase difference between the two recording beams at λ_j_ and the spatial periodicity of the corresponding light pattern, while θ is the incident angle of the beams (Figure 1a). The first term describes the interference pattern produced by parallel-polarized (PP) lines, contributing to the overall field through the combination of sinusoidal intensity distributions sharing a same polarization state, identical to that of the incident recording beams. χ_j_ denotes the ellipticity and ψ_j_ the azimuthal angle of the polarization ellipse, while I_j_ indicates the intensity associated with the j-th line. A schematic representation of the light pattern is reported in the first line of the scheme reported in the right part of Figure 1a. The second term corresponds to the interference arising from orthogonally circular (OC) polarized beams. Here, the patterns exhibit spatially uniform intensity distribution and a modulated polarization state, namely, the polarization is linear everywhere with the azimuthal angle which is periodically spatially modulated through a continuous rotation (according to the polarization pattern reported in second line of the scheme in Figure 1a). The third term corresponds to the interference arising from orthogonally linear (OL) polarized beams, where α and α + π/2 represent the orientation angles of the linearly polarized electric fields relative to the *y*-axis (as shown in Figure 1a). Here the interference field amplitude remains spatially uniform, although the polarization exhibits spatial modulation of the ellipticity (according to the polarization pattern reported in third line of the scheme in Figure 1a).

## 3. Surface Photo-Morphing of Complex Hierarchical Design

Thin films of azopolymers are excellent substrates for demonstrating the potential of this technique in achieving direct and reversible surface structuring. These materials respond to spatially patterned UV/visible light through cyclic transitions between trans and cis isomeric forms. This photoinduced isomerization drives mass transport within the polymer, leading to changes in surface topography. The final surface morphology is determined by the spatial and spectral characteristics of the illuminating light pattern, which control the depth, shape, and direction of the overall deformation.

An azobenzene amorphous side-chain polymer, specifically Poly-Disperse Red 1 Methacrylate (pDR1Ma, CAS:139096-37-0, Sigma Aldrich, Darmstadt, Germany), is chosen due to its nearly uniform absorption across all laser wavelengths [35]. The ground-state trans-azobenzene readily isomerizes to the cis form under wavelength-selective irradiation. This photoinduced process is completely reversible, allowing the trans state to be restored through appropriate light exposure or heating.

Before exposure, the chromophores are randomly oriented, rendering the film optically isotropic. When irradiated with suitable wavelengths, they undergo continuous trans-cis-trans photoisomerization cycles until their axes reorient perpendicularly to the light polarization direction, making the film anisotropic.

Films with a thickness of approximately 600 nm were prepared by spin coating a solution of pDR1Ma (7% *w*/*v* in chloroform) onto a glass substrate.

To demonstrate the capability of the method to operate fully within the deterministic spectrum of Fourier and hierarchical topography engineering, we investigated surface morphing following exposure to the three encoding schemes of Equation (1): an intensity pattern with linear parallel polarization (PP), and the polarization patterns of opposite circular (OC) and orthogonal linear (OL) configurations, Figure 1a. We selected a beam spectral composition comprising four lines of equal intensity to clearly visualize the method’s capabilities in terms of fidelity and hierarchical connections between different scales.

In the first geometry of PP light patterns, the configuration with polarization lying in the plane of incidence (pPP) has proven to be the most effective for surface relief grating (SRG) inscription using monochromatic intensity or amplitude patterns [36,37]. In this setup, the electric field is linearly polarized along the direction of the grating wavevector, and the photoinduced mass migration arises from the combined action of polarization-induced anisotropy and local intensity gradients. Several physical mechanisms have been proposed to account for the driving force behind the observed topographical modifications, including thermal, pressure, and permittivity gradients, as well as optical forces associated with the photoinduced anisotropy of the material [38,39,40]. A common observation across these studies is the migration of material toward the less illuminated regions, resulting in SRGs where surface relief minima correspond to the maxima of light intensity.

The OC configuration generates a polarization pattern with continuously rotating linear polarization and an almost uniform amplitude (with a Gaussian profile) across the irradiated area (Figure 1a). In contrast, the OL configuration produces a polarization pattern characterized by a modulation of ellipticity from linear to circular, and an azimuthal angle that alternates between ±π⁄4 relative to the recording linear polarizations (Figure 1a). These geometries highlight the vectorial nature of the photoinduced processes contributing to topographical deformation while minimizing the occurrence of additional phenomena related to intensity modulation.

The OC encoding scheme is well known as the most efficient for SRG formation in azomaterials that exhibit only linear photoinduced birefringence, such as side-chain azopolymers. However, in materials exhibiting both linear and circular photoinduced anisotropies, the OL recording geometry can enhance the complexity of the topography and the resolution of the structures [41].

It is worth mentioning that the processes involved in SRG formation induced by polarization patterns are complex, and a coherent theoretical framework capable of accurately modeling the photoinduced effects still remains a subject of debate [40,41,42].

To enable a consistent comparison, we generated interference patterns using laser beams composed of four spectral lines, each adjusted to deliver the same amplitude (Figure 1b), while keeping both the total irradiance and exposure time constant. All samples were irradiated for 360 s using two beams, each with an optical power of 25 mW, as measured by a Nova II power meter (Ophir). The beams intersected at an angle of (29 ± 1)°, producing interference fringes with spatial periods of approximately 1 μm, as reported in the corresponding table in Figure 1b. The spectral composition of the interfering beams was analyzed using a fiber-coupled spectrometer (AvaSpec-ULS2048, Avantes, Apeldoorn, Netherlands) with a spectral resolution of 2 nm. A linearly polarized He-Ne laser (25-LHP-151, Melles Griot, Carlsbad, California, USA), operating at 632.8 nm, outside the absorption range of the polymer [35], was used to monitor the development of the surface relief during exposure. Two linear CCD photodiode arrays (LC1-USB 2.0, Thorlabs, Newton, New Jersey, USA) were placed along the transmitted beam path to acquire the corresponding far-field diffraction profiles (Figure 1b).

**Figure 2:**
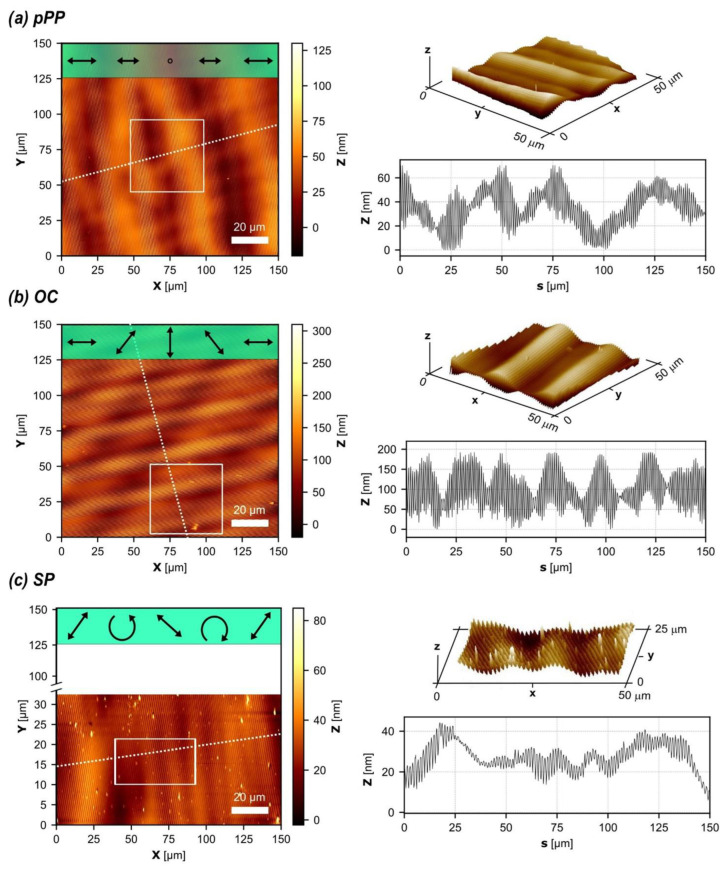
AFM analysis of the surface topography. (**a**) pPP configuration: the investigation involves an area of 150 × 150 μm^2^ (2D map); the line square indicates the zone displayed as 3D detail (right side). The 1D profile is recovered following the dotted line. (**b**) OC configuration: the investigation involves an area of 150 × 150 μm^2^ (2D map); the line square indicates the zone displayed as 3D detail (right side). The 1D profile is recovered following the dotted line. (**c**) OL (sp) configuration: the investigation involves an area of 35 × 150 μm^2^ (2D map); the line square indicates the zone displayed as 3D detail (right side). The 1D profile is recovered following the dotted line.

Figure 2 presents the AFM surface topographies and corresponding one-dimensional height profiles obtained after photopatterning under pPP, OC, and OL configurations. The surface morphology of the photo-patterned films was characterized at room temperature using a Bruker Multimode 8 atomic force microscope, using PeakForce mode and controlled via a Nanoscope V system. Imaging was performed with silicon cantilevers (RTESPA-300, Bruker, Billerica, Massachusetts, USA) featuring a nominal spring constant of 42 N/m and a tip radius of approximately 10 nm. The scan rate and sampling density (points per line) were optimized based on the selected scan area to ensure accurate topographic resolution.

In all cases, the complex structuring of the free surface is observed. A large-scale relief modulation is superimposed on small-scale ridges with an about 1 µm periodicity and amplitude varying irregularly from a few nanometers to several hundred nanometers.

These multiscale surfaces exhibit multiple scales in both the height and width of their morphology. As expected for azomaterials displaying linear photoinduced anisotropy, the OC configuration is more efficient than the OL configuration, showing greater relief depth at both small and large scales, as well as a more pronounced sinusoidal profile. In contrast, the surface photopatterned with the pPP configuration exhibits a less regular profile compared to the others.

The fast Fourier transform (FFT) is a crucial tool for processing such surface data, allowing the decomposition of a patterned surface into its harmonic frequencies, the detection of hierarchical structures, and the identification and quantification of their characteristics.

**Figure 3:**
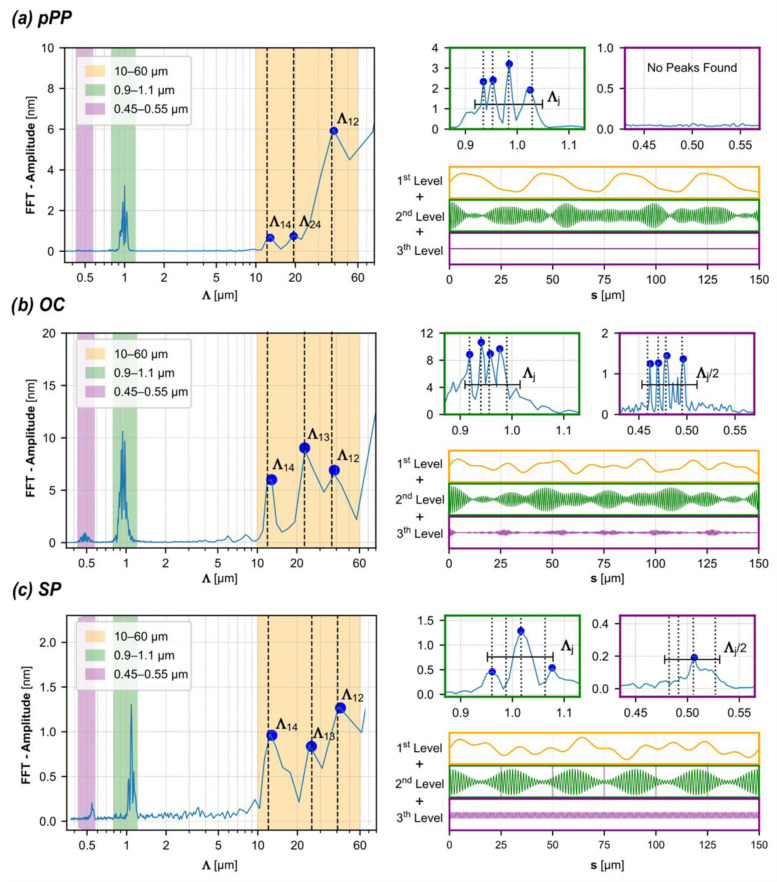
FFT analysis of the 1D profiles shown in Figure 2. The FFT spectra highlight characteristic length scale ranges: violet (0.45–0.55 µm), green (0.9–1.1 µm), and orange (10–60 µm), corresponding to distinct structural features. Details of the small-scale spectra are enlarged in the right-hand column. Below each spectrum, the additive contribution of the structural levels is shown. Analysis of surfaces generated by (**a**) pPP photopatterning, (**b**) OC photopatterning, and (**c**) OL (sp) photopatterning.

Figure 3 presents the FFTs of the surface profiles, which clearly reveal the fundamental spatial periodicities (Λj) of the recorded structures at the expected positions, with amplitudes that accurately replicate the recording compositions, demonstrating the high sensitivity and resolution of the technique. Notably, laser lines with wavelengths separated by only a few nanometers (e.g., λ_3_ and λ_4_) enable the recording of well-distinguished structures with high fidelity.

Additionally, the FFT shows other contributions at both lower and higher spatial periods. In the case of OC and OL photopatterning, some peaks appear in the smaller spatial periodicity range (around 500 nm). These peaks correspond to half of the encoding polarization pattern periods. While these half-period structures are expected for OL (s-p polarized) photopatterning [43,44], they are not for OC. In the latter case, the contribution at half-periodicity can be attributed to the interference field of the first diffracted orders generated during the encoding process within the material’s volume, due to their polarization characteristics and high efficiency [45]. During the patterning process, as the bulk structure forms, self-diffraction of the recording beams occurs. The two first diffraction orders, one for each recording beam, are oppositely circularly polarized and interfere at an incidence angle of 2θ with respect to the *z*-axis. Notably, these smaller-scale structures are absent in the pPP photopatterning, where the scalar encoding and the growth processes cannot account for the formation of periodic structures at ($\Lambda_{j}/2).$

Peaks with larger spatial periodicities (>10 µm) are clearly observed in all cases, with some differences in amplitude compared to the fundamental ones. They exhibit comparable amplitudes for the topographies recorded with polarization patterns, Figure 3b,c. Although a comprehensive study on the topographical structuring of azomaterials under multi-wavelength interference is still ongoing, the experimental findings provide some interesting insights.

In particular, the measured large scale spatial periodicities of the surface morphology can be explained by considering beat phenomena arising from spatially modulated optomechanical stresses, which mediate the combination of the $\Lambda_{j}$ anisotropy modulations to produce the overall topography. Specifically, their spatial periods ($\Lambda_{nm}$) can be determined by calculating the beats between the different $\Lambda_{j}=\lambda_{j}/2\;sin\theta$ pairs (couples) as follows:

(2) $$\Lambda_{nm}=\lambda_{n}\lambda_{m}/2\left|\lambda_{n}-\lambda_{m}\right|sin\theta$$

**Figure 4:**
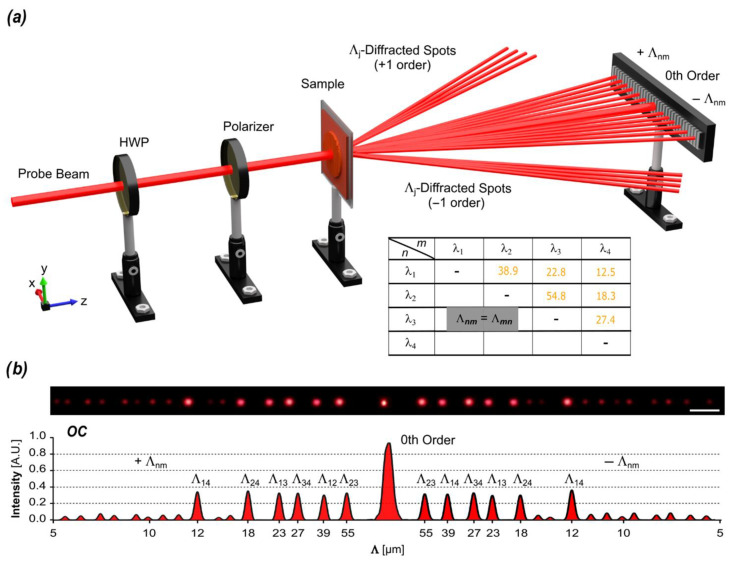
Far-field diffraction pattern of light from the OC photopatterned azopolymer film. (**a**) Schematic representation of the experimental setup used to measure the far-field diffraction pattern. A linearly polarized probe beam passes through the sample, producing multiple diffraction orders that are collected on a screen. The resulting diffraction orders correspond to spatial periods Λ_j_ and Λ_nm_. (**b**) Far-field diffraction pattern generated by the large-scale periodic surface structures, along with the corresponding intensity profile. The table shows the calculated spatial periods Λ_nm_ resulting from the superposition of different primary Λ_j_ structures.

These spatial periodicities, an order of magnitude higher than the fundamental ones, are listed in the table in Figure 4a. Some of them, specifically the lower ones, are directly visible in the FFT spectra. However, the higher values (i.e., Λ_23_) do not appear in the FFT analysis of the profiles (Figure 3). This is due to the limitations of the AFM acquisition area [150 × 150] µm^2^, which restricts the measurement of larger spatial periodicities because the scanned surface area is not large enough to capture the full diffraction pattern associated with these periodicities.

Nevertheless, by examining the far-field diffraction pattern produced by a probe laser beam transmitted through the film, which is effectively the Fourier transform of the encoded structures, all the expected spatial periodicities can be easily identified Figure 4b. Light spots of nearly equal intensity, corresponding to diffraction angles attributable to the expected large-scale spatial periodicities, are clearly visible in the far field. Moreover, these diffracted beams emerge several seconds after those corresponding to the fundamental periodicities, supporting the hypothesis of the occurrence of the beat phenomena as free surface oscillations induced by bulk-modulated stresses.

Additionally, the same set of experiments performed on azopolymer films confined between glass substrates (which inhibit SRG formation) do not produce this large-scale diffraction contribution.

The experimental findings reveal that three levels of hierarchical morphologies emerge ($\Lambda_{j}/2$, $\Lambda_{j}$, $\Lambda_{nm}$), all of which are deterministic, since they are related to the intensity and the spectral composition of the interfering light fields, as shown in Figure 3. These levels combine additively. In such a kind of hierarchy, the hierarchical surface is defined as the sum of the heights of the involved levels, with specific weights quantifying their interaction. In this case, the weights are equal, given the identical intensity values of the four laser lines and the comparable absorption of the material within their wavelength range.

To further investigate the essence of hierarchy and to quantitatively assess the characteristics and complexity of the surfaces induced by this vectorial holographic approach, we employed several methodological tools, as reported in refs. [46,47]. These methods were chosen because they allow for a comprehensive analysis of surface morphologies, providing insights into both the regularity and complexity of the surface structures formed, as well on the interaction between the processes involved at different scales.

Specifically, we focused on the following two key aspects of symmetry: translational symmetry, which describes the regular repetition of surface features in space, and scaling symmetry, which characterizes how morphological features repeat across different length scales. These features were quantified by their periodicity and fractal dimension, respectively.

**Figure 5:**
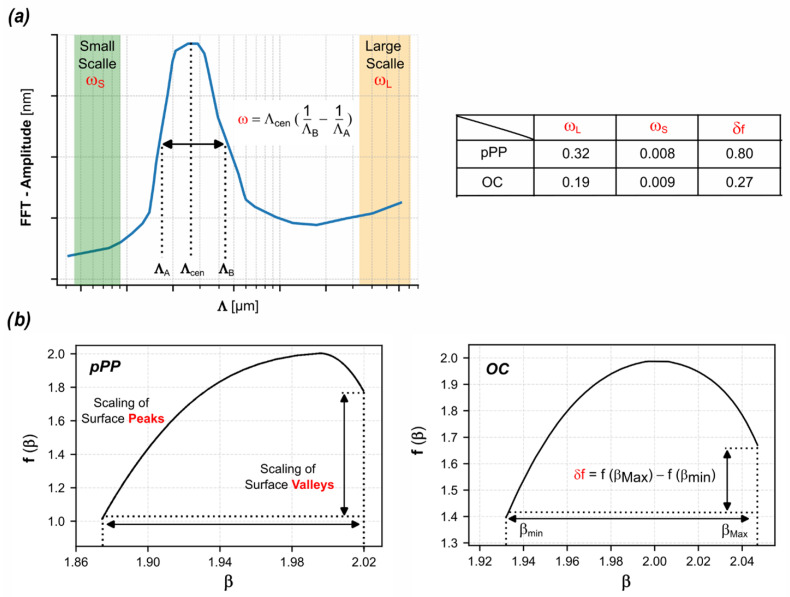
Analysis of the surfaces structural complexity. (**a**) Schematic representation of the ω parameter, which quantifies the peak width in the FFT spectra at two distinct spatial scales: small (S), for Λ_j_, and large (L) for Λ_nm_. A representative FFT peak is shown, with the regions used for the ω calculation highlighted in color, in green for ω_S_ and in orange for ω_L_. (**b**) Illustration of the scaling disorder parameter δf, obtained from multifractal analysis, which reflects the range of surface height fluctuations and the asymmetry in scaling behavior between peaks and valleys. Summary of the values of the parameters ω and δf for surfaces patterned using pPP and OC interference configurations are reported in the accompanying table.

To detect deviations from these symmetries, referred to as translational and scaling disorder, we employed the following two key metrics: (I) Translational disorder which was quantified using the full width at half maximum (FWHM) of the peaks in the Fourier spectrum, Figure 5a. A larger FWHM indicates greater deviation from perfect periodicity. (II) Scaling disorder, which was assessed through the multifractal spectrum, capturing the complexity of the scaling behavior and revealing the presence of multiple scaling exponents, Figure 5b.

Fractal geometry is a very useful tool when complex surfaces are under investigation. By the term complex, we mean surfaces that contain structures at different sizes/scales and density. The coexistence of a number of scales and fractal symmetries, sometimes combined in nonlinear ways on the surface, makes it even harder to distinguish. Multifractal (MF) analysis is an extension of the fractal geometry used in order to bypass these difficulties. The MF spectrum is a parabola-like spectrum of exponents which is called a singularity spectrum *f*(α). It is obtained with the box counting method, which measures the scaling behavior of a surface versus surface heights, while differentiating the fractal analysis of the top from bottom parts of the surface. In the MF spectrum, the *x*-axis (*α*) handles the height segmentation (peak region *α < 2*, to valley region *α > 2*), while the *y*-axis contains the fractal exponents corresponding to the value of *α, f(α).* Using the spectrum, we are able to separate the behavior of the surface peaks (left branch of the MF parabola) from the bottom areas of the surface (right branch of the MF parabola). The width of the spectrum *δα* indicates the vertical range of the surface fluctuations and the horizontal *δf = f*(*α_max_*) *− f*(*α_min_*) reveals a common measurement of the multifractality, meaning that it essentially quantifies the asymmetry between the scaling behavior of the top and bottom part of the surface.

These metrics not only allowed us to detect the presence of disorder but also to classify its type and extent, providing a tool for deeper understanding of the hierarchical nature and complexity of the surface morphologies obtained with the present vectorial holographic approach.

We focused on the surfaces that exhibit the most effective structuring, specifically the ones recorded using the intensity pPP light pattern and the ones recorded using the OC polarization pattern. We evaluated the translational disorder parameters (ω_L_ and ω_S_) for both large- and small-scale peaks, Figure 5a, (namely the ones at $\Lambda_{nm}$ and $\Lambda_{j}$), and the scaling disorder parameter (δf, Figure 5b) from multifractal analysis, which assesses the range of surface fluctuations and the difference in scaling behavior between surface peaks and valleys.

The deviation from perfect periodicity, quantified by the translational disorder (the ω_s_ parameters) is very low (practically negligible) for $\Lambda_{j}$. Specifically, we obtained ω_S_ = 0.008 for pPP photopatterning and ω_S_ = 0.009 for OC photopatterning. This indicates that the structures straightforward related to the primary process, directly triggered by the light, are efficiently and faithfully replicated on the surface. In contrast, for the large-scale structures, the ω_L_ values increase by more than one order of magnitude compared to the ω_S_ values, reaching ω_L_ = 0.32 for pPP and ω_L_ = 0.19 for OC. This suggests a higher degree of disorder in the large-scale structures, which likely arises from the additive processes of light-induced viscoplastic deformations in the material. Additionally, a greater degree of disorder is observed in the structures recorded by the intensity pattern. Similarly, the scaling disorder parameter δf decreases from 0.80 for the pPP photopatterned surfaces to 0.27 for the OC photopatterned surfaces, indicating that the OC patterning results in a more regular hierarchical morphology with reduced scaling complexity. The overall results indicate that the translational behavior, reflecting deviations from perfect periodicity, and the scaling behavior of the upper and lower segments of the structured surfaces are more symmetrical for the hierarchical topographies generated by OC photopatterning than for those produced by the pPP pattern. This observation highlights the significant impact of photo-morphing geometries and the associated processes on surface symmetry and disorder characteristics.

The observed difference between the two approaches suggests that the spatial modulation of light intensity, characteristic of scalar encoding fields (pPP), introduces noise effects in hierarchical structuring, either attenuating or amplifying surface deformations. Specifically, in pPP patterns, regions of high illumination are immediately adjacent to dark regions, unlike OC patterns where the intensity distribution is uniform along the grating period [45]. These sharp intensity gradients can lead to localized variations in temperature, density, and viscosity within the material, triggering additional processes that overlay the optically induced mechanical stresses and randomly influence the resulting surface topography. These findings demonstrate that the choice of encoding scheme (OC vs. pPP) plays a critical role in determining the symmetry, regularity, and hierarchical complexity of the resulting surface topographies. This understanding offers a pathway for precise control over the morphological characteristics of photoresponsive substrates.

Furthermore, when the photoresponsive substrate is based on a photosensitive material whose photoresponse involves reversible processes (such as photoisomerization in our case), the surface morphology can be dynamically modified simply by adjusting (or resetting) the optical structure and the spectral composition of the interference field, thus offering the opportunity to dynamically and remotely control not only the complexity but also the degree of disorder of the surface morphology.

## 4. Conclusions

Interference-based light patterning remains a versatile and powerful technique for material structuring, with emerging functionalities that extend beyond its traditional applications [48]. This study demonstrates that employing a polychromatic laser source, an unconventional approach for holographic encoding, unveils new collective phenomena and enables the deterministic fabrication of hierarchical Fourier surfaces through a straightforward and reproducible method.

Even within the simplest two-beam interference geometry, broadband vectorial holography allows the superposition of independent stress modulations at multiple spatial scales, producing unique surface features via beat effects. These effects lead to previously unreported hierarchical topographical structures in azobenzene-containing materials, characterized by multiscale periodicities ranging from submicron to hundreds of microns. The reversibility of photoinduced effects in azomaterials suggests the possibility of the dynamic control and reconfiguration of surface morphologies.

The scalability and adaptability of this approach make it suitable for more complex interference schemes, including multi-beam configurations, which provide additional degrees of freedom for surface patterning [49].

Combined with the intrinsic advantages of optical interference lithography, this method presents a competitive strategy for the rapid, high-precision fabrication of compact surface architectures. Potential applications span photonics and optics, imaging technologies, advanced material design, biomimetics, information security, and anti-counterfeiting solutions.

## Data Availability

The data of this study are available from the corresponding author upon reasonable request.
